# Transmission of Syphilis from the Father

**Published:** 1880-10

**Authors:** 


Transmission of Syphilis from the Father.—Wolf, of Strasburg, has studied a large number of syphilitic parents and their children, and reaches the following conclusions:

The transmission of pox from the father to his progeny can only occur through infection of the mother.

In every case of a child being born with syphilis, the mother is or has been syphilitic. Wolf has never seen a single case in which he was not able to verify this proposition.

It is easy to see the importance of this assertion, both theoretically and practically, particularly in reference to natural nursing.

